# Green tea and type 2 diabetes mellitus

**Published:** 2014-01-01

**Authors:** Mahmoud Rafieian-Kopaei, Parisa Motamedi, Leila Vakili, Nahid Dehghani, Fereshte Kiani, Zahra Taheri, Sara Torkamaneh, Parto Nasri, Hamid Nasri

**Affiliations:** ^1^Medical Plants Research Center, Shahrekord University of Medical Sciences, Shahrekord, Iran; ^2^Environmental Health Engineering, Engineering Department, Health Faculty, Isfahan University of Medical Sciences, Isfahan, Iran; ^3^Nour Medical Hospital, Isfahan University of Medical Sciences, Isfahan, Iran; ^4^Young Researchers and Elite Club, Isfahan (Khorasgan) Branch, Islamic Azad University, Isfahan, Iran; ^5^Department of Biostatistics and Epidemiology, School of Health, Isfahan University of Medical Sciences, Isfahan, Iran; ^6^Social Health Determinants Research Center, Shahrekord University of Medical Sciences, Shahrekord, Iran; ^7^Department of Physical Education and Sport Sciences, Khorasgan University of Isfahan, Isfahan, Iran; ^8^Department of Nephrology, Isfahan University of Medical Sciences, Isfahan, Iran

**Keywords:** Green tea, Renal injury, Nephrotoxicity, Diabetes mellitus

**Implication for health policy/practice/research/medical education:**

Diabetic individuals may benefit from kidney protective efficacies of green tea, as well as its blood glucose regulatory impacts. Additionally, it should be noticed that, green tea may present a nontoxic, effective and inexpensive modality as a supplementary substance in diabetic patients.

Tea is the most common beverage throughout the world. Green tea (Camellia sinensis) is full of flavonoids (1). This herbal drug has a lot of polyphenols, catechin epicatechin, epigallocatechin and epigallocatechin-3-gallate. It also contains other composites like saponins, caffeine, tannins, and some vitamins. Several different investigations have indicated that green tea has hypo-cholesterolaemic, antioxidant, antimutagenic and anti-inflammatory actions. This is mostly due to the impact of galic acid and catechin provided in green tea (1,2). Various studies have shown that, amongst the flavonoids that are contained in green tea, polyphenol type of tannins and catechins may be acquired. Among these epicatechin, epicatechin-3-gallate, catechins, epigallocatechin and epigallocatechin-3-gallate are the prevalent types which are consisted in green tea. Epigallocatechin-3-gallate has been revealed to have a preventive and treatment influence on various types of chronic diseases along with its anticancer properties (2-6). Additionally some studies have also shown that catechin presented in green tea inhibits the proliferation of breast cancer cells (6-8).

Type 2 diabetes mellitus is the most common and on the rise chronic metabolic disease, globally (3,4).

Clinical investigations have detected that in type II diabetic patients, oxidative stress plays an important role in producing the adverse effects of diabetes. Rise in free radicals causes strengthen in peroxidation of lipids and increase in insulin resistance (1-4). Green tea contains flavonoids and various polyphenols which have anti-inflammatory and anti-oxidative property (4-6). Recently to find the influence of green tea consumption on metabolic and anthropometric parameters of patients with type II diabetes, Mossavi et al., investigated the possible efficiency of various daily doses of green tea consumption for eight weeks on various anthropometric, metabolic, and oxidative stress biomarkers of diabetic individuals (7). The randomized clinical trial was conducted on 63 type II diabetes patients. They found that consumption of four cups of green tea per day produced a significant decrease in body mass index, body weight, waist circumference and systolic blood pressure (7). Green tea also directed to a significant reduction in weight and systolic blood pressure (7). It is clear that reactive oxygen species are mediators of renal damage and green tea is also a potent free radical scavenger (2-5). To examine whether green tea could protect against the renal damage induced by contrast media, we previously conducted a study on 40 rats, which were randomly divided into four groups including: 1) control group, 2) contrast media group, 3) contrast media plus green tea, 4) contrast media and green tea pretreatment group. Histological and biochemical changes regarding the severity of renal injury were assessed (8). In this investigation, beneficial efficacy of green tea against renal tubular toxicity of contrast media by examination of kidney function and structure (8). In diabetic nephropathy tubular damage by various mechanisms is a part of this disease (3-5). Thus, green tea is capable of protecting the renal tubular cells from oxidative stress in diabetic patients, beyond its beneficial effects on metabolic and anthropometric indices in patients with type 2 diabetes (2,7,8). Likewise Mousavi et al. conducted investigations to evaluate the influence of green tea consumption on components of metabolic syndrome in the elderly. They found the positive efficacy of green tea in inducing weight loss, waist circumference and reducing body mass index in elderly patients with metabolic syndrome (7). Additionally, Mozaffari-Khosravi et al. examined the ameliorative influence of sour tea and green tea on blood pressure of patients with type II diabetic patients by a randomized clinical trial on 100 mildly hypertensive individuals. They detected that mildly hypertensive type II diabetic patients who drank three glasses of sour tea or green tea, daily for 4 weeks, had a significant decrease in systolic and diastolic blood pressures (9). Hence, consumption of green tea for the treatment of hypertension may further protect kidney in diabetic individuals (1-6). In diabetic nephropathy, the glomeruli have been at the focus of notice as the primary site of damage in diabetic kidney disease. On the contrary, it is well known that tubulointerstitial changes are notorious components of the disease, particularly in individuals with type II diabetes (2-6). In fact, proteinuria and diabetic nephropathy progression are finely associated with interstitial fibrosis tubular injury (3-6). Actually, in the process of diabetic nephropathy, capillary rarefaction directed to local ischemia with additional injury to the tubules, further profibrogenic mediator of fibrosis, matrix protein deposition and finally intensification of the glomerulosclerosis (3-6). Therefore, in diabetic kidney disease, the tubules harbor changes that are frequently correlated with glomerular alterations, tubular apoptosis, tubular cell degeneration and tubular atrophy (6-9). Thus, it is reasonable to illuminate that green tea has two different efficacies; an ameliorative effect on metabolic and anthropometric indices as well as level of blood pressure which indirectly protect the kidney against nephropathy of diabetes and secondly tubular protection by acting as an effective antioxidant. Therefore, diabetic individuals may benefit from both of these two distinct efficacies, as well as its blood glucose regulatory impacts. Additionally, it should be noticed that green tea may present a nontoxic, effective and inexpensive modality as a supplementary substance in diabetic patients (8-11).

## Authors’ contributions

All authors contributed to the paper equally.

## Conflict of interests

The authors declared no competing interests.

## Ethical considerations

Ethical issues (including plagiarism, misconduct, data fabrication, falsification, double publication or submission, redundancy) have been completely observed by the authors.

## Funding/Support

None.
